# Decitabine and Melphalan Fail to Reactivate p73 in p53 Deficient Myeloma Cells

**DOI:** 10.3390/ijms19010040

**Published:** 2017-12-23

**Authors:** Pierre-Samuel Gillardin, Géraldine Descamps, Sophie Maiga, Benoit Tessoulin, Hanane Djamai, Benedetta Lucani, David Chiron, Philippe Moreau, Steven Le Gouill, Martine Amiot, Catherine Pellat-Deceunynck, Agnès Moreau-Aubry

**Affiliations:** 1 CRCINA, INSERM, CNRS, Université d’Angers, Université de Nantes, 44007 Nantes, France; pierre.gillardin@etu.univ-nantes.fr (P.-S.G.); geraldine.descamps@univ-nantes.fr (G.D.); sophie.maiga@inserm.fr (S.M.); benoit.tessoulin@chu-nantes.fr (B.T.); djamai.hanane@gmail.com (H.D.); benedetta.lucani@gmail.com (B.L.); david.chiron@inserm.fr (D.C.); philippe.moreau@chu-nantes.fr (P.M.); steven.legouill@chu-nantes.fr (S.L.G.); martine.amiot@inserm.fr (M.A.); agnes.aubry@univ-nantes.fr (A.M.-A.); 2 Service d’Hématologie Clinique, Unité d’Investigation Clinique, CHU, 44093 Nantes, France

**Keywords:** multiple myeloma, p53, p73, CpG methylation, decitabine, alkylating drugs

## Abstract

(1) Background: *TP53* deficiency remains a major adverse event in Multiple Myeloma (MM) despite therapeutic progresses. As it is not possible to target *TP53* deficiency with pharmacological agents, we explored the possibility of activating another p53 family member, p73, which has not been well studied in myeloma. (2) Methods: Using human myeloma cell lines (HMCLs) with normal or abnormal *TP53* status, we assessed *TP73* methylation and expression. (3) Results: Using microarray data, we reported that *TP73* is weakly expressed in 47 HMCLs and mostly in *TP53* wild type (*TP53^wt^*) HMCLs (*p* = 0.0029). Q-RT-PCR assays showed that *TP73* was expressed in 57% of *TP53^wt^* HMCLs (4 out of 7) and 11% of *TP53* abnormal (*TP53^abn^*) HMCLs (2 out of 18) (*p* = 0.0463). We showed that *TP73* is silenced by methylation in *TP53^abn^* HMCLs and that decitabine increased its expression, which, however, remained insufficient for significant protein expression. Alkylating drugs increased expression of *TP73* only in *TP53^wt^* HMCLs but failed to synergize with decitabine in *TP53^abn^* HMCLs. (4) Conclusions: Decitabine and melphalan does not appear as a promising combination for inducing p73 and bypassing p53 deficiency in myeloma cells.

## 1. Introduction

Multiple Myeloma (MM) is characterized by an important biological heterogeneity related to recurrent chromosomal abnormalities, which occurs early in the disease development at the MGUS (Monoclonal Gammopathy of Undetermined Significance) stage [1]. The recurrent chromosomal abnormalities are IgH gene translocation with recurrent partners located on chromosomes 4, 6, 11, 16 and 20 or hyperdiploidy of odd chromosomes [1]. Different prognoses and overall survivals are associated with this first heterogeneity. However, and independently from this heterogeneity, the most adverse prognosis is related to chromosomal deletion of *TP53* [2]. Mutations of *TP53* were exclusively found in myeloma cells displaying a hemi-deletion of the short arm of chromosome 17 (del17p) [3]. The frequency of *TP53* mutations, which is low at diagnosis (around 3%), increases with relapses and is high in plasma cell leukemia (PCL, >30%) and human myeloma cell lines (HMCLs, >70%), these latter mostly deriving from extramedullary MM and mainly from PCL [4,5,6,7,8]. Loss of function of mutant p53 is believed to be related to conformation deficiency: therefore, small molecules, such as RITA and Prima-1^Met^, which were selected for their ability to induce cell death in p53 mutated cells were shown to bind to p53 protein [9]. However, in our hands, both molecules induced massive cell death but their efficacy was unrelated to *TP53* status and p53 expression [10,11]. Because del(17)p and *TP53* mutations, which are irreversible, are an adverse event whatever the treatment regimen, alternative treatments that bypass or circumvent p53 are needed. For instance, the p53 family member p73 could be of interest as p73 is also able to transactivate pro-apoptotic genes [12,13]. Moreover, *TP73* is rarely mutated but frequently silenced by CpG methylation in hematological malignancies and in multiple myeloma (MM) [14,15,16,17]. *TP73* expression might thus be reactivated by drugs preventing CpG methylation after DNA replication such as decitabine. To assess the interest in activating p73 in myeloma cells, we studied p73 expression and regulation, *TP73* methylation and *TP73* sequencing in a large panel of human myeloma cell lines HMCLs with a normal or abnormal *TP53* status [7,18].

## 2. Results

### 2.1. TP73 Is Preferentially Constitutively Expressed in TP53^wt^ HMCLs 

**Figure 1:**
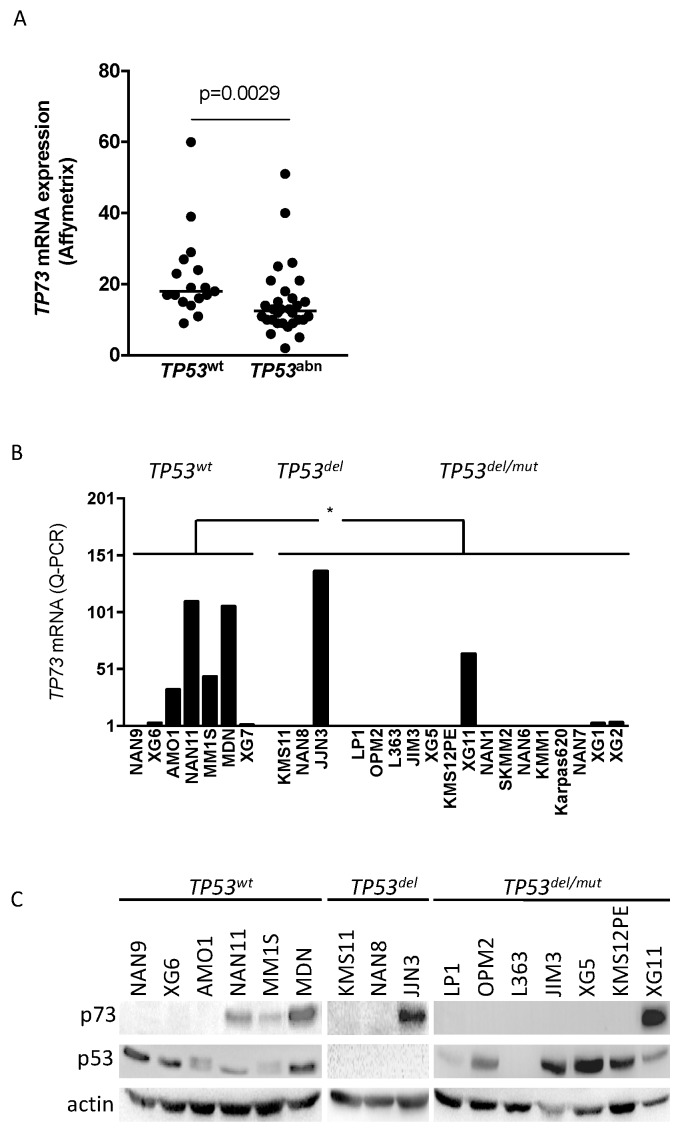
Constitutive *TP73* expression was mainly restricted to *TP53^wt^* HMCLs: (**A**) Constitutive expression of *TP73* in 47 HMCLs was performed by microarray (Affymetrix) and analyzed according to *TP53* status. (**B**) Constitutive expression of *TP73* was mainly restricted to *TP53^wt^* HMCLs. Expression was assessed by Q-PCR in 25 HMCLs. The histograms represent the mean ± SEM of 3 experiments (SEM fall within the symbols). *, *p* < 0.05. (**C**) Constitutive expression of p73 was mainly restricted to *TP73*^+^ HMCLs. Expression of p73 and p53 was assessed by Western blotting with anti-p73 and anti-p53 antibodies.

We assessed expression of *TP73* mRNA in 47 HMCLs using Affymetrix microarray data [7]. Although expression level was very low, HMCLs with normal *TP53* status (*TP53^wt^*) appeared to significantly express higher level of *TP73* mRNA than *TP53^abn^* HMCLs (*p* = 0.0029, Mann–Whitney test, Figure 1). To confirm this observation, we further selected 25 HMCLs displaying either a normal (*n* = 7) or an abnormal *TP53* status (*n* = 18, 3 *TP53* deleted (*TP53^del^*) and 15 *TP53 mutated* (*TP53^mut^*) HMCLs) (Table 1) [7,11,18]. Status of *TP53* and *TP73* was determined by sequencing of RT-PCR products and/or whole exon sequencing. Q-RT-PCR assays showed that *TP73* was expressed in four out of seven *TP53^wt^* HMCLs (AMO1, MDN, MM1S, NAN11), in one out of three *TP53^del^* HMCLs (JJN3) and in one out of fifteen *TP53^mut^* HMCLs (XG11), *p* = 0.0463, Mann–Whitney test, (Figure 1B). p73 expression was confirmed at the protein level using Western blotting for MDN, MM1S, NAN11, JJN3 and XG11 but not for AMO1 (Figure 1C). Whole exon sequencing showed that only KMM1 and XG11 displayed a missense mutation (A211S and I626V, respectively). However, XG11, but not KM11, expressed *TP73* and p73. The strong p73 expression in XG11 might be related to the presence of the mutation within the transactivation inhibitory domain, which was reported to be involved in p73 degradation [19].

### 2.2. Decitabine Decreased TP73 Methylation and Induced TP73 Expression 

**Figure 2:**
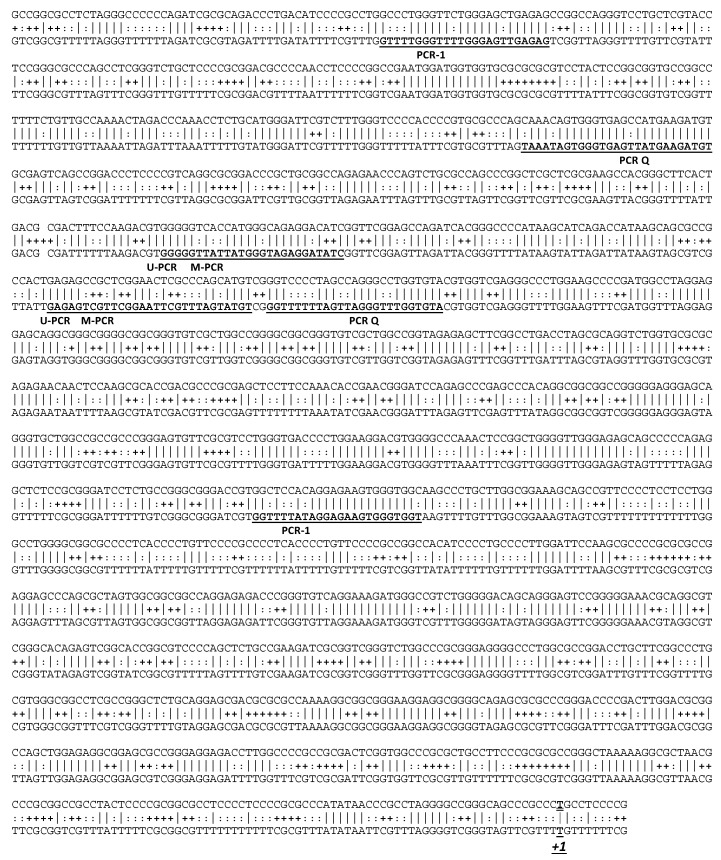
Targeting of CpG islands in *TP73*. Upper and lower sequences represent the native and bisulfite converted *TP73* DNA sequence, respectively. The CpG are indicated with “+”. Primers used for PCR1, M-PCR (Methylated-PCR), U-PCR (Unmethylated methylation) and PCR-Q (Quantifying PCR) are indicated by underlines. *+1* indicates the first transcripted nucleotide.

**Figure 3:**
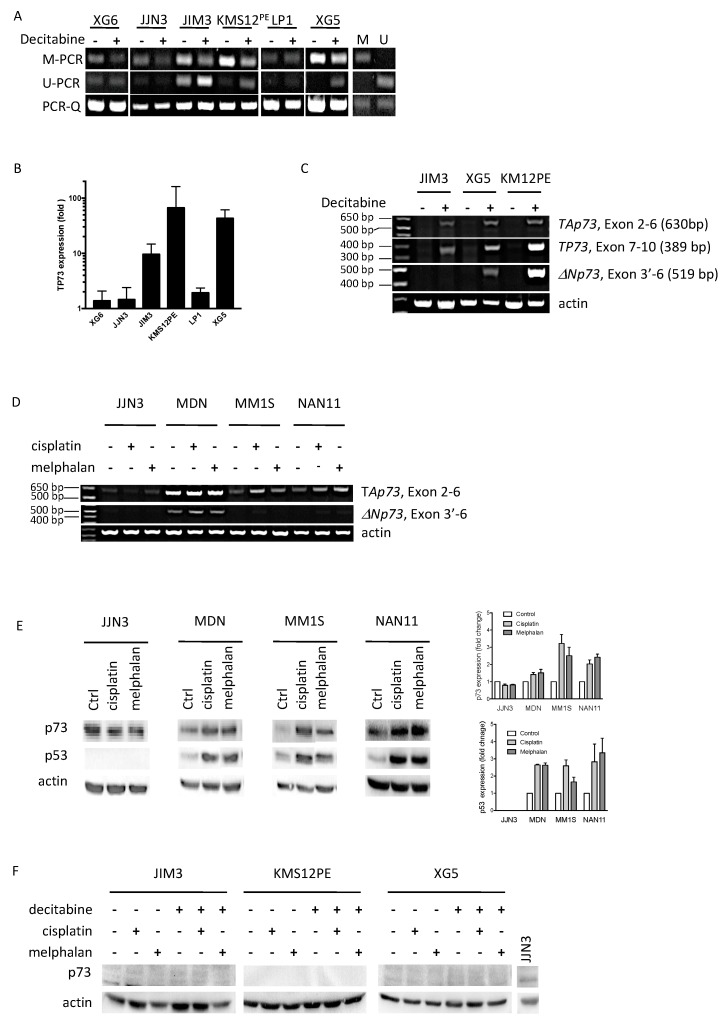
Cisplatin or melphalan induced the expression of *TAp73* isoform in *TP53^wt^* HMCLs but not in *TP53^abn^* HMCLs. (**A**) *TP73* is mainly methylated in *TP53^mut^* HMCLs. MS-PCRs were performed after bisulfite conversion of DNA extracted from HMCLs treated or not with decitabine (5 µM, 72 h). M and U indicated methylated and unmethylated control DNAs. (**B**) Decitabine induced *TP73* expression. *TP73* expression was assessed by Q-PCR, as described in the legend of Figure 1. (**C**) Decitabine induced expression of several *TP73* isoforms. RT-PCRs were run on cDNAs from control or decitabine-treated HMCLs (5 µM, 72 h), as indicated in the figure. (**D**) Cisplatin or melphalan increased the expression of *TAp73* isoform. RT-PCR expression of *TAp73* and Δ*Np73* was performed after a 16-h treatment with melphalan or cisplatin. *TP53^wt^* HMCLs were treated with 7 μM of melphalan or cisplatin, JJN3 cells were treated with 30 μM of melphalan or 20 μM of cisplatin. (**E**) Cisplatin or melphalan increased the expression p73 in *TP53^wt^* HMCLs. Western blots were performed after an overnight treatment with melphalan or cisplatin as described in (**A**). Right part represents the quantification of p73 and p53 over actin expression from two independent experiments (mean ± SEM). (**F**) Decitabine and melphalan or cisplatin failed to induce p73 expression in *TP53^mut^* HMCLs. HMCLs were treated 72 h with decitabine (5 μM) prior to an overnight treatment with 45 µM of melphalan or 55 µM of cisplatin. JJN3 proteins were loaded as a control of p73 expression.

To assess the methylation level in *TP73* promoter, we performed nested methyl-specific PCR (MS-PCR) targeting the CpG island upstream of the gene (Figure 2). PCR-1 was run on bisulfite converted DNA with primers that did not involve CpG. The PCR-1 product was then used for three nested PCRs; two, respectively, specific of unconverted CpG (methylated PCR, M-PCR) and converted CpG (unmethylated PCR, U-PCR) sequence; and one quantifying the PCR-1 product (PCR-Q, Figure 2). PCRs were run in HMCLs treated or not with decitabine to assess the *TP73* methylation status (Figure 3A). In parallel, we measured the expression level of *TP73* by Q-PCR (Figure 3B). In JIM3, KMS12PE and XG5, *TP73* was found methylated and decitabine induced a decrease in M-PCR and an increase in U-PCR, which correlated with an increase in *TP73* expression. However, despite the increase in *TP73* expression, we could not detect any protein. Because anti-p73 Ab was directed against the N terminal part of the protein (amino acids 1–62) and could thus not detect ΔNp73, we performed three RT-PCRs to assess the total *TP73* expression (exons 7–10), the full-length TAp73 (exons 2–6) and the Δ*Np73* (exons 3′–6) isoforms, respectively. The *TAp73* isoform was increased in the three *TP53^mut^* HMCLs, although in KMS12PE the Δ*Np73* isoform was the main increased isoform (Figure 3C). The data suggested that the lack of p73 detection by Western blotting was not related to the lack of *TAp73* transactivation, but rather to a weak transactivation. 

### 2.3. Melphalan and Cisplatin Induced TP73 Expression in TP53^wt^ HMCLs but Not in Decitabine-Treated TP53^mut^ HMCLs

To further study *TP73* regulation, we treated four HMCLs that constitutively expressed p73 with alkylating drugs, melphalan and cisplatin and determined by RT-PCR and Western blotting the regulation of *TP73* and p73. Regulation of p53 expression was assessed as a control of drug response [20]. Alkylating drugs increased the expression of the p73 full-length isoform in the *TP53^wt^* MDN, MM1S and NAN11 HMCLs but not in TP53 deleted (*TP53^del^*) JJN3 HMCL (Figure 3D). MDN also expressed the Δ*Np73* isoform that was slightly increased by the drugs. By Western blotting, we confirmed that melphalan and cisplatin similarly increased p73 expression in the three *TP53^wt^* HMCLs but failed to increase p73 expression in *TP53^del^* JJN3 (Figure 3E). As expected, p53 expression was increased by both drugs in the *TP53^wt^* MDN, MM1S and NAN11 HMCLs but not in *TP53^del^* JJN3 cells [20].

We then assessed whether melphalan could increase expression of p73 in decitabine-treated *TP53^mut^* HMCLs. We selected the three HMCLs that displayed a strong increase in *TP73* expression upon decitabin treatment, i.e., JIM3, KMS12PE and XG5 (Figure 3B). As shown in Figure 3F, alkylating drugs failed to increase any p73 expression that remained undetectable in the 3 *TP5*3*^mut^* HMCLs. 

## 3. Discussion

In myeloma, regulation of p73 expression has not been deeply investigated yet. For instance, *TP73* expression has been shown to be induced by PRIMA-1^Met^ in several HMCLs including JJN3 in which p73 was reported to be partly involved in PRIMA-1^Met^-induced cell death [17,21]. However, we did not confirm these results in JJN3, suggesting that JJN3 might be misidentified between the different laboratories. In hematological malignancies, *TP73* that does not display frequent mutations is known to be silenced by CpG methylation [14]. Indeed, whole exon sequencing showed that only two HMCLs (KMM1 and XG11) harbored a mutation in *TP73* gene. We thus assessed *TP73* methylation and regulation in a large number of HMCLs. Our results showed that *TP73* is mainly silenced by methylation and that the methylation inhibitor decitabine reversed the methylation-mediated silencing. However, the *TP73* expression remained too weak for allowing a detectable p73 expression by Western blotting. We showed that decitabine-induced expression of both *TAp73* and Δ*Np73* isoforms: Δ*Np73*, which is lacking the transactivation domain, is known to inhibit TAp73 binding to DNA [19]. In KMS12PE, the high decitabine-induced expression of Δ*Np73* isoform might explain the lack of p73 detection by Western blotting because the anti-p73 Abs was directed against the N terminal part of the protein (aa 1-62). *TP73* gene also harbors an intrinsic CpG island that governs Δ*Np73* isoform transcription. Thus, *TP73* gene appears particularly methylated in MM cells and demethylation might equally induce expression of *TAp73* and Δ*Np73* isoforms, the latter being an inhibitory isoform [19]. We found that p73 was constitutively expressed in six HMCLs: MDN, MM1S, NAN11 and XG10 expressed a wildtype p53 protein, JJN3 lacked p53 expression and XG11 expressed a mutant p53 protein. Of note, p73 was overexpressed in XG11 despite a moderate *TP73* expression suggesting a stabilization/lack of degradation of the protein that might be related to the presence of the mutation in the transactivation inhibitory domain, which is involved in degradation [22]. In three out of four HMCLs that constitutively expressed p73 and displayed an unmethylated profile of CpG, alkylating drugs increased both *TP73* and p73 expression: this regulation seemed to be restricted to *TP53^wt^* HMCLs as it did not occur in the *TP53^del^* JJN3 HMCL. This finding is consistent with the possible regulation of *TP73* expression via p53/p21/E2F1, which can only occur in *TP53^wt^* HMCLs [23]. Indeed, alkylating drugs were unable to increase p73 expression in the three *TP53^mut^* HMCLs that displayed a *TP73* expression after decitabine treatment. Moreover, MDM2, which stabilizes p73 instead of inducing its degradation [24], could take a part in the p73 increase induced by alkylating drugs, because MDM2 is a p53 target gene mostly expressed in *TP53^wt^* HMCLs [18]. Nevertheless, the role of p53 in melphalan-induced p73 regulation, if any, requires further investigations. On the other hand, *TP73* expression was shown to be regulated by HDAC: indeed, the HDAC inhibitor sodium butyrate increased the “free” E2F1 pool and therefore *TP73* transactivation independently from p53 [25]. We did not investigate the HDAC-mediated regulation of p73 in myeloma cells but it would be of interest to study HDAC inhibitors in combination with decitabine in p53 deficient cells. In summary, our data show that decitabine and melphalan does not appear as a promising combination for bypassing p53 deficiency in myeloma cells.

## 4. Materials and Methods 

### 4.1. HMCLs and Reagents

HMCLs were previously described [7,11]. Unmethylated/methylated control DNAs, and the bisulfite conversion kit were purchased from Active Motif (La Hulpe, Belgium). Anti-p73 A300-126A and anti-p53 DO-1 Abs were purchased from Bethyl Laboratories (Euromedex, Souffelweyersheim, France) and Millipore (Saint-Quentin en Yvelines, France), respectively. Anti-actin was purchased from Millipore. Cisplatin and melphalan were purchased from Sigma Aldrich (Saint-Quentin-Fallavier, France). Quantitative PCR probes were purchased from Taqman (Thermofisher, Saint-Herblain, France). Quantitative RT-PCR assays were performed as previously described [7].

### 4.2. Bisulfite Treatment, Methylation Specific PCRs and RT-PCR

DNAs were treated with bisulfite as indicated by the supplier. Methylation specific PCRs (M-PCR, U-PCR) and quantifying PCR (PCR-Q) were performed after a first amplification (PCR1). PCR1, M-PCR, U-PCR and PCR Q were run with 25, 17, 20 and 19 cycles, respectively. The following primers were used: PCR-1 GTTTTGGGTTTTGGGAGTTGAGAG and ACCACCCACTTCTCCTATAAAA (874 bp; M-PCR GGGGTTATTATGGGTAGAGGATATC and ACATACTAAACGAATTCCGAACGACTC (109 bp); U-PCR GGGTTATTATGGGTAGAGGATATT and ACATACTAAACAAATTCCAAACAACTCTC (112 bp); and PCR Q TAAATAGTGGGTGAGTTATGAAGATGT and TACACCAAACCCTAACTAAAAAACC (285 bp). Extracted RNAs were reversed transcripted and amplified as previously described [12]. 

For *TP73* RT-PCR assays, the following primers were used: *TAp73* ex2-6 CACCACGTTTGAGCACCTCT and AGATTATTGCCTTCCACGCG (630 bp); *TP73* ex7-10 GACGGAATTCACCACCATCCT and CCAGGCTCTCTTTCAGCTTC (389 bp); and Δ*Np73* ex3′-6 CCATGCTGTACGTCGGTGAC and CCAAATCCTTCTCCCTATCC (519 bp). For *TP73* Q-PCR assays, the *TP73* (Hs01056230_m1) and *RPL37A* (Hs01102345_m1) probes were used.

### 4.3 Western Blotting

Expression of p53 and p73 was determined by Western blotting as previously described [11]. A minimum of 80 μg of proteins was loaded in each lane. 

## Figures and Tables

**Table 1:** Human myeloma cell lines (HMCL) characteristics.

HMCL		*TP53* Status and Expression		*TP73* Status and Expression		
Name	Translocation	*TP53*	p53	*TP73*	Q-PCR	p73
AMO1	unknown	wt	+	wt	+	−
MDN	(11;14)	wt	+	wt	+	+
MM1S	(14;16)	wt	+	wt	+	+
NAN9	(4;14)	wt	+	wt	−	−
NAN11	(14;16)	wt	+	wt	+	+
XG6	(16;22)	wt	+	wt	+/−	−
XG7	(4;14)	wt	+	wt	+/−	nd
JJN3	(14;16)	deletion	−	wt	+	+
KMS11	(4;14)	deletion	−	wt	−	−
NAN8	(4;14)	disrupted ^1^	−	wt	−	−
JIM3	(4;14)	R273C	+	wt	−	−
Karpas620	(11;14)	C135Y	+	wt	−	nd
KMM1	(6;14)	C135F	+	A211S	−	nd
KMS12PE	(11;14)	R337L	+	wt	−	−
LP1	(4;14)	E286K	+	wt	−	−
L363	(20;22)	S261T ^2^	−	wt	−	−
NAN1	(14;16)	E180STOP	−	wt	−	nd
NAN6	(14;16)	Indel ^3^	+ ^5^	wt	−	nd
NAN7	(11;14)	Indel ^4^	−	wt	−	nd
OPM2	(4;14)	R175H	+	wt	−	−
SKMM2	(11;14)	K132N	+	wt	−	nd
XG11	(11;14)	C135Y	+	I626V	+	+
XG1	(11;14)	Y126N	+	wt	+/−	nd
XG2	unknown	C176Y	+	wt	+/−	nd
XG5	(11;14)	R282W	+	wt	−	−

nd: not done; ^1^ disrupted by amplification of exons 1, 2, 3, 4, 5 and 6; ^2^ lack of intron 7 splicing; ^3^ deletion of exons 7, 8 and 9; ^4^ deletion of exon 11; ^5^ truncated form.

## Abbreviations

HMCL	Human Myeloma Cell Line
MM	Multiple Myeloma
MS-PCR	Methylation-specific PCR
